# The Incremental Value of Repeated Induced Sputum and Gastric Aspirate Samples for the Diagnosis of Pulmonary Tuberculosis in Young Children With Acute Community-Acquired Pneumonia

**DOI:** 10.1093/cid/cix099

**Published:** 2017-05-27

**Authors:** David P. Moore, Melissa M. Higdon, Laura L. Hammitt, Christine Prosperi, Andrea N. DeLuca, Pedro Da Silva, Vicky L. Baillie, Peter V. Adrian, Azwifarwi Mudau, Maria Deloria Knoll, Daniel R. Feikin, David R. Murdoch, Katherine L. O’Brien, Shabir A. Madhi

**Affiliations:** 1 Medical Research Council, Respiratory and Meningeal Pathogens Research Unit,; 2 Department of Science and Technology/National Research Foundation, Vaccine Preventable Diseases Unit, and; 3 Department of Clinical Microbiology & Infectious Diseases, University of the Witwatersrand,; 4 Department of Paediatrics & Child Health, Chris Hani Baragwanath Academic Hospital and University of the Witwatersrand, Johannesburg, and; 5 Mycobacteriology Referral Laboratory, National Health Laboratory Service, Braamfontein, South Africa;; 6 Department of International Health, International Vaccine Access Center, Johns Hopkins Bloomberg School of Public Health, and; 7 Department of Epidemiology, Johns Hopkins Bloomberg School of Public Health, Baltimore, Maryland;; 8 Division of Viral Diseases, National Center for Immunizations and Respiratory Diseases, Centers for Disease Control and Prevention, Atlanta, Georgia;; 9 Kenya Medical Research Institute-Wellcome Trust Research Programme, Kilifi;; 10 Department of Pathology, University of Otago, and; 11 Microbiology Unit, Canterbury Health Laboratories, Christchurch, New Zealand

**Keywords:** child, tuberculosis, induced sputum, gastric aspirate, yield.

## Abstract

**Background.:**

*Mycobacterium tuberculosis* (*Mtb*) contributes to the pathogenesis of childhood acute community-acquired pneumonia in settings with a high tuberculosis burden. The incremental value of a repeated induced sputum (IS) sample, compared with a single IS or gastric aspirate (GA) sample, is not well known.

**Methods.:**

Two IS samples were obtained for *Mtb* culture from children enrolled as cases in the Pneumonia Etiology Research for Child Health (PERCH) study in South Africa. Nonstudy attending physicians requested GA if pulmonary tuberculosis was clinically suspected. We compared the *Mtb* yield of 2 IS samples to that of 1 IS sample and GA samples.

**Results:**

. Twenty-seven (3.0%) culture-confirmed pulmonary tuberculosis cases were identified among 906 children investigated with IS and GA samples for *Mtb*. Results from 2 IS samples were available for 719 children (79.4%). Of 12 culture-confirmed pulmonary tuberculosis cases identified among children with ≥2 IS samples, 4 (33.3%) were negative at the first IS sample. In head-to-head comparisons among children with both GA and IS samples collected, the yield of 1 GA sample (8 of 427; 1.9%) was similar to that of 1 IS sample (5 of 427, 1.2%), and the yield of 2 GA samples (10 of 300; 3.3%) was similar to that of 2 IS samples (5 of 300; 1.7%). IS samples identified 8 (42.1%) of the 19 culture-confirmed pulmonary tuberculosis cases that were identified through submission of IS and GA samples.

**Conclusions.:**

A single IS sample underestimated the presence of *Mtb* in children hospitalized with severe or very severe pneumonia. Detection of *Mtb* is enhanced by combining 2 IS with GA sample collections in young children with acute severe pneumonia.

The optimal approach to diagnosis of childhood pulmonary tuberculosis remains to be determined, as existing methods lack sensitivity [1]. In settings with a high tuberculosis burden, up to 15% of incident tuberculosis cases occur in children <14 years of age. Also, children <5 years of age are at higher risk of developing disease soon after infection with *Mycobacterium tuberculosis* (*Mtb*), most often manifest as pulmonary tuberculosis [2].

The microbiological diagnosis of pulmonary tuberculosis depends on detection of *Mtb* in respiratory samples obtained from a suspected case. Because children <5 years of age swallow rather than expectorate their sputum, the recommended approach to obtain respiratory secretions is by gastric aspirate (GA) or induced sputum (IS) sample collection [3]. The acidic environment in the stomach, however, may impair the viability of *Mtb* in GA samples, leading to lower yields compared with other respiratory samples [4]. Because primary pulmonary tuberculosis in children is generally noncavitating, results of microscopy of respiratory samples for acid-fast bacilli are often negative, and the sensitivity of culture of respiratory secretion samples among clinically diagnosed pulmonary tuberculosis cases is 7%–53% [5–8].

The feasibility of using IS sampling as an alternate to GA sampling for culturing *Mtb* was demonstrated in South African children <5 years of age hospitalized with clinically suspected pulmonary tuberculosis [9]. The same study reported that *Mtb* culture positivity from a single IS sample was similar to that from 3 GA samples collected on consecutive days [9]. A more recent systematic literature review and meta-analysis estimated that 1–3 IS samples may identify 79% (95% confidence interval [CI], 62%–92%) of culture-confirmed pediatric pulmonary tuberculosis cases identified through submission of comparator samples, including GA, pleural/lymph node culture, and nasopharyngeal aspirate samples [10].

Whereas pulmonary tuberculosis is generally considered a subacute or chronic illness [11–13], studies from high-burden countries have demonstrated that up to 8% of children hospitalized for acute community-acquired pneumonia also have culture-confirmed tuberculosis [14–16]. It remains unclear, however, whether pulmonary tuberculosis diagnosed in young children with severe acute community-acquired pneumonia represents *Mtb* as a cause of the pneumonia, or whether the pulmonary tuberculosis is an underlying immunosuppressive condition predisposing children to acute bacterial or viral pneumonia [16].

The objective of the current study was to compare the yield of a single IS sample with that of 2 IS samples collected on separate days for culture of *Mtb* in children <5 years of age hospitalized with severe or very severe community-acquired pneumonia, in a setting with a high tuberculosis and human immunodeficiency virus (HIV) burden. In addition, we compared the *Mtb* culture yield of 1 or 2 IS samples to the yield from 1, 2, or 3 GA samples obtained as part of standard care in these children.

## METHODS

### Cases

Children between 1 and 59 months of age hospitalized with World Health Organization (WHO)–defined severe or very severe pneumonia were recruited as part of the Pneumonia Etiology Research for Child Health (PERCH) study at the South African site, based at Chris Hani Baragwanath Academic Hospital, Soweto, Gauteng Province from 17 August 2011 to 31 August 2013. Exclusion criteria for cases were hospitalization within the previous 14 days, discharge as a PERCH case within the past 30 days, residence outside the study catchment area, or resolution of lower chest wall indrawing after bronchodilator therapy for those with wheezing. Further details about case identification and case definitions are provided elsewhere [17, 18].

IS samples were obtained for standard microbiological and *Mtb* culture in the children enrolled into the PERCH study, as well as polymerase chain reaction testing for multiple respiratory pathogens [19]. Whereas all the PERCH sites planned on collecting at least a single IS sample from the cases, it was decided a priori at the South African site to collect 2 IS samples on separate days to compare the yield of *Mtb* culture between 1 and 2 IS samples. The second IS sample was obtained for study purposes only, and not as part of the standard of care. In 2011 and 2012, the incidence (per 100 000 population) of a hospital-based diagnosis of culture-confirmed pulmonary tuberculosis in children <5 years of age resident in Soweto was 33 (95% CI, 26–41), and 185 (114–282) in HIV-positive children [20]. Because of the high tuberculosis burden at the South African site [20–23], clinicians admitting children with pneumonia frequently send 2 or 3 GA samples on separate days for *Mtb* culture. The vertical transmission prevalence of HIV infection from mother to child at the community level has been <3% in Gauteng Province since 2010, and the prevalence of HIV infection among children hospitalized at Chris Hani Baragwanath Academic Hospital was 19% in 2010/2011 [24, 25].

### Procedures

Collection of IS samples was undertaken according to a standardized operating procedure used by all PERCH sites [26]. Trained study nurses obtained most of the IS samples, including those collected from children admitted to the high-care ward, where the procedure was performed under supervision of the study clinician. Whenever possible, the first IS sample was obtained on the morning after enrollment, and the second obtained 24–72 hours after the initial sample. All IS sample collections were undertaken at least 2–3 hours after the child’s most recent meal or feeding. Temporary contraindications to IS sample collection have been described elsewhere [27].

IS samples were transported in a cooler box to the research laboratory for processing. The first sample was apportioned into 3 aliquots, 1 each for molecular testing, standard microbiological culture, and *Mtb* culture. The second IS sample was processed only for *Mtb* culture.

GA samples were collected after an overnight fast of ≥4 hours in a subset of PERCH cases with suspected *Mtb* infection, at the discretion of treating clinicians. Hospital procedure is to collect 3 GA samples on 3 consecutive days, whenever possible, to evaluate young children with suspected pulmonary tuberculosis. GA samples were collected into sterile containers without sodium bicarbonate buffer.

IS aliquots (designated for *Mtb* culture) and GA samples were sent to the National Health Laboratory Service Mycobacteriology Referral Laboratory, Braamfontein, Johannesburg. Samples were processed according to current guidelines by decontamination and digestion with sodium hydroxide/N-acetyl-cysteine [28]. Fluorescent microscopy for mycobacteria was conducted by auramine O staining of a portion of the resuspended sputum/aspirate pellet. Samples were inoculated into mycobacterial growth indicator vials (MGIT; Becton-Dickinson) primed with growth supplement and antibiotics (PANTA™; Becton-Dickinson), which were incubated for a maximum of 42 days. Positive cultures were examined microscopically for acid-fast bacilli staining positive with Ziehl-Neelsen stain, which were identified using a commercially available line probe assay (HAIN GenoType *Mtb*DRplus, HAIN Lifescience). Flag-positive MGIT vials that stained negative for acid-fast bacilli and lacked contaminants were reincubated. Flag-positive MGIT vials that on staining demonstrated the presence of contaminants only were not processed further.

Children clinically suspected of having pulmonary tuberculosis were tested using tuberculin skin tests, administered according to the Mantoux method by nonresearch clinicians [29]. Tuberculin skin tests were interpreted 48–72 hours after intradermal inoculation of 0.5 mL of Tuberculin PPD RT 23 (Statens Serum Institut), according to WHO guidelines [3].

Participant discharge diagnoses were abstracted from health records, and the hospital-based tuberculosis registry was reviewed to determine whether pulmonary tuberculosis had been diagnosed clinically by the attending clinician during the hospitalization. A case with clinically diagnosed tuberculosis was defined as a child who, regardless of tuberculin skin test response, had antituberculosis treatment started and was registered as having pulmonary tuberculosis during the PERCH hospitalization episode but had negative *Mtb* cultures. Children were designated as having culture-confirmed pulmonary tuberculosis if the IS, GA or other samples (endotracheal tube aspirates and mycobacterial blood cultures) were culture positive for *Mtb*. Children with nonrespiratory specimens (eg, *Mtb* cultured on blood) were deemed to have pulmonary tuberculosis if chest radiographic findings suggested pulmonary infection.

### Analysis

We hypothesized that comprehensiveness of respiratory specimen sampling to establish a diagnosis of pulmonary tuberculosis in acutely ill children may be influenced by the severity of illness and the degree of clinical suspicion of pulmonary tuberculosis. We conducted univariate and multivariate analyses, using forward stepwise logistic regression, to determine which factors were associated with collection of 1 rather than 2 IS samples, as well as factors associated with the treating clinician’s decision to request GA sample collection. Factors associated with collection of 2 IS samples and those associated with GA sample collections were incorporated into the multivariate models if 2-sided *P* values were ≤0.20 at univariate analysis.

The yield of culture-confirmed *Mtb* was assessed in 7 comparisons: 1 versus 2 IS samples, 1 IS versus 1 GA sample, 1 IS versus 2 GA samples, 1 IS versus 3 GA samples, 2 IS versus 1 GA sample, 2 IS versus 2 GA samples, and 2 IS versus 3 GA samples. McNemar χ^2^ testing was performed to test the difference in yield between paired samples. Children who did not have IS or GA samples available for pairwise comparison according to the schema set out for comparison analyses outlined above, were excluded as appropriate. Occasionally, IS and GA samples were submitted for auramine O staining for mycobacteria and were not processed further for mycobacterial culture. Only IS or GA samples with available mycobacterial culture results were included in these analyses. Children with smear-positive, culture-negative samples were not considered confirmed pulmonary tuberculosis cases [30].

Contamination of IS and GA samples with oropharyngeal bacterial or fungal organisms may inadvertently occur during sample collection or processing and thus represents an actual operational scenario [31, 32]. Focusing our analysis only on instances where contaminated specimens did not occur would tend to overestimate the utility of IS and GA in pediatric pulmonary tuberculosis diagnosis (by decreasing sample denominators while maintaining the numerator). Our analysis therefore included contaminated specimens in the denominators of IS and GA submitted to identify the culture-confirmed pulmonary tuberculosis cases. All analyses were performed using Stata version 13.0 (StataCorp, College Station, TX). Permissions to conduct the study, nested within the overarching PERCH study, were obtained from the institutional review boards of the University of the Witwatersrand and the Johns Hopkins Bloomberg School of Public Health.

## RESULTS

### Baseline Characteristics

Of 920 children enrolled as cases with WHO-defined severe (n = 622; 67.6%) or very severe (n = 298; 32.4%) pneumonia at the South African PERCH site, 906 (98.5%) had at least one IS or GA collected: 840 (92.7%) of these had at least one IS sample collected, with a second sample obtained in 785 (86.6%). Furthermore, 399 (44.0%) cases had ≥2 GA samples collected, on consecutive days in 228 (57.1%) of the 399, >1 day apart in 122 (30.6%), and on the same day in 49 (12.3%). Of the 2782 samples submitted to the laboratory, 157 (5.6%) did not undergo *Mtb* culture for varying reasons (75 culture not requested, 34 lost in transit to the laboratory, 22 leaked, 22 unlabeled, 4 not processed owing to laboratory accidents). The first IS sample result was available in 799 children, of whom 328 (41.1%) had 1 IS and ≥2 GA results, and 300 (37.5%) had 2 IS and ≥2 GA results (see Supplementary Figure 1).

Indicators of increasing pneumonia severity (central cyanosis at baseline and in-hospital death) were the 2 factors that, at multivariate analysis, were associated with omission of a second IS sample collection among children who had 1 IS sample submitted (data not shown). There were significant differences in baseline characteristics between children with and those without a GA sample collected. At multivariate analysis, GA sample collection was positively associated with increasing age, duration of difficulty breathing, and positive tuberculin skin test result and negatively associated with nasal flaring and administration of antituberculosis treatment on the day of enrollment (Supplementary Table 1).

Of the 920 cases with pneumonia, 146 (15.9%) were identified either clinically or by culture confirmation as having pulmonary tuberculosis, 119 (12.9%) were discharged with a clinical diagnosis of pulmonary tuberculosis without culture confirmation, and 27 (2.9%) had culture-confirmed tuberculosis, including 1 with a diagnosis based on mycobacterial blood culture whose chest radiograph was abnormal and whose 2 IS cultures were negative (case 27 in Table 1). Twenty-five (92.6%) of the 27 children with culture-confirmed pulmonary tuberculosis were subjected to IS sample collections, and 19 (70.4%) had GA samples submitted. Similarly, 114 (95.8%) of the 119 children with clinically diagnosed pulmonary tuberculosis had IS samples submitted, and 97 (81.5%) underwent GA sample collections. The crude yields of *Mtb* culture positivity in IS and GA samples among the children with clinically diagnosed and/or culture-confirmed pulmonary tuberculosis were 9% (13 of 139) and 14% (16 of 116), respectively. Forty-eight (32.9%) of the 146 children with pulmonary tuberculosis were HIV positive, 3 with culture-confirmed and 45 with clinically diagnosed pulmonary tuberculosis. HIV-positive children had a 6-fold greater odds (95% CI, 3.96–10.11) of having clinically diagnosed pulmonary tuberculosis but were just as likely to have culture-confirmed pulmonary tuberculosis as those who were HIV negative (odds ratio, 0.87; 95% CI, .17–2.94).

**Table 1.** Culture-Confirmed Pulmonary Tuberculosis Cases in the South African PERCH Cohort^a^

Case	HIV Status	Pneumonia Severity	CR	IS Sample		GA Sample			ETT Sample		*Mtb* Blood Culture	Samples With Pos *Mtb* Culture	Basis for Diagnosis					Added Value Analysis^b^
				1st	2nd	1st	2nd	3rd	1st	2nd			GA Only (n = 27)	IS Only (n = 27)	GA + IS (n = 27)	Other Sample (n = 27)	IS in Cases With GA Samples (n = 19)	
1	Neg	Very severe	AC	**Neg** ^**c**^	**Neg**	**Pos**	**Neg**	N/R^1^				GA1	✓					1–3, 5, 6
2	Neg	Severe	Uninterp	**Neg**	**Neg**	**Pos**	**Neg**	Neg				GA1	✓					1–7
3	Neg	Severe	Normal	**Neg**	**Neg**	**Pos**	**Neg**	Neg				GA1	✓					1–7
4	Neg	Severe	AC	**Neg**	**Neg**	**Pos**	**Neg**	N/R^2^				GA1	✓					1–3, 5, 6
5	Neg	Very severe	Normal	Neg	Neg	Pos						GA1	✓					1, 2, 5
6	Neg	Severe	AC	**Neg**	**Neg**	**Neg**	**Pos**	Neg				GA2	✓					1–7
7	Pos	Very severe	AC	**Neg**	**Neg**	**Neg**	**Pos**					GA2	✓					1–3, 5, 6
8	Neg	Severe	Normal	**Neg**	**Neg**	**Neg**	**Pos**	Neg				GA2	✓					1–7
9	Neg	Severe	AC	N/R^2^	Neg	Neg	Pos	Pos				GA2, GA3	✓					Excluded^d^
10	Neg	Severe	AC	Neg	Neg	N/R^3^	Neg	Pos				GA3	✓					1
11	Pos	Severe	AC	Neg	Neg	Neg	N/R^2^	Pos				GA3	✓					1, 2, 5
12	Neg	Severe	Normal	**Pos**	**Neg**	**Neg**	**Neg**	Neg				IS1		✓			✓	1–7
13	Neg	Very severe	AC	Pos	Neg							IS1		✓				1
14	Neg	Severe	AC	**Pos**	**Neg**	**Neg**	**Neg**					IS1		✓			✓	1–3, 5, 6
15	Neg	Very severe	Normal	Pos	Neg							IS1		✓				1
16	Neg	Very severe	AC	Pos	Pos	Neg						IS1, IS2		✓			✓	1, 2, 5
17	Neg	Severe	AC	Neg	Pos							IS2		✓				1
18	Neg	Severe	Normal	Neg	Pos							IS2		✓				1
19	Neg	Severe	Normal	Neg	Pos							IS2		✓				1
20	Neg	Very severe	AC	**Pos**	**Neg**	**Pos**	**Neg**					IS1, GA1			✓		✓	1–3, 5, 6
21	Neg	Severe	AC	Pos	N/R^4^	N/R^4^	Pos					IS1, GA2			✓		✓	Excluded^d^
22	Neg	Severe	AC	Pos	Neg	N/R^1^	Pos	Pos				IS1, GA2, GA3			✓		✓	1
23	Neg	Severe	AC	**Pos**	**Pos**	**Pos**	**Pos**					IS1, IS2, GA1, GA2			✓		✓	1–3, 5, 6
24	Neg	Severe	AC	**Neg**	**Pos**	**Pos**	**Neg**	Neg	Neg			IS2, GA1			✓		✓	1–7
25	Neg	Very severe	AC						Pos	Neg		ETT1				✓		
26	Pos	Very severe	AC						Neg	Pos		ETT2				✓		
27	Neg	Severe	AC	Neg	Neg						Pos	*Mtb* blood culture				✓		1
Total, No. (%)													11 (40.7)	8 (29.6)	5 (18.5)	3 (11.1)	8 (42.1)	

Abbreviation AC, alveolar consolidation; CR, chest radiograph; ETT, endotracheal tube sputum sample; GA, gastric aspirate; HIV, human immunodeficiency virus; IS, induced sputum; *Mtb*, *Mycobacterium tuberculosis*; Neg, negative; N/R, not resulted; N/R^1^, specimen leaked; N/R^2^, culture not requested; N/R^3^, unlabeled specimen; N/R^4^, smear positive, culture not requested; PERCH, Pneumonia Etiology Research for Child Health; Pos, positive; Uninterp, uninterpretable.

^a^Children were designated as having culture-confirmed pulmonary tuberculosis if they had a specimen positive for *Mtb* at mycobacterial culture, in the presence of an abnormal chest radiograph. All but 1 of the 27 cases with culture-confirmed pulmonary tuberculosis had their illness confirmed through culture of respiratory specimens (GA, IS, or ETT). One child had a positive *Mtb* blood culture in the presence of an abnormal chest radiograph. The table is sorted by culture-confirmed pulmonary tuberculosis cases, according to type of specimen that was positive for *Mtb* on culture, listing GA before IS samples, then GA- and IS-positive cases, followed by cases culture positive for *Mtb* from specimens other than GA and IS.

^b^See Figure 1

^c^Bolded cells highlight the following: in the 2-IS and 2-GA sample comparison in which *Mtb* culture results were available, the first IS sample identified 4 (33.3%) of the 12 positive pulmonary tuberculosis cases, and the GA samples with or without the second IS sample identified the remaining 8 (66.7%).

^d^Children were excluded from the added yield analyses if mycobacterial culture results were unavailable for the first IS sample (case 9) or the first GA and second IS samples (case 21).

**Figure 1.**
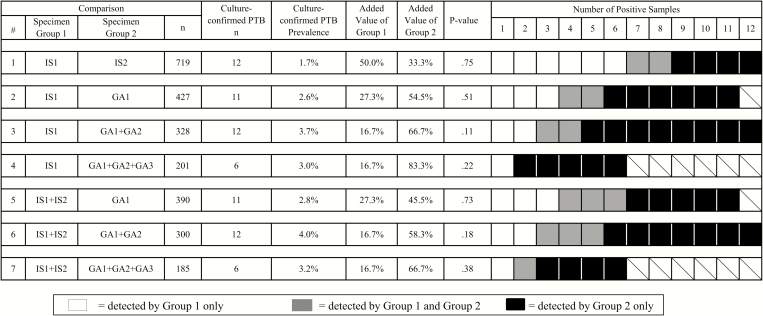
Added value of induced sputum (IS) and gastric aspirate (GA) specimens in the detection of *Mycobacterium tuberculosis* (*Mtb*). Added value represents the number of cases positive for *Mtb* that were detected by submission of specimens/specimen groups and not by the comparator specimens/specimen groups. *P* values were determined with exact McNemar tests. Boxes with diagonal lines represent categories not applicable for comparison.

Auramine O staining was performed on samples from 896 (98.9%) of 906 children with samples submitted for mycobacterial culture. Eight (0.9%) had microscopic findings positive for mycobacteria, including 2 with culture-confirmed *Mtb*, 1 with clinically diagnosed pulmonary tuberculosis without culture confirmation, and 5 in whom a clinical diagnosis of pulmonary tuberculosis was not made.

Contamination of IS samples occurred in 47 (5.9%) and 64 (8.6%) of first and second IS samples, respectively, with higher contamination rates for the second IS samples (odds ratio, 1.50; 95% CI, 1.00–2.27). Contamination rates ranged from 5.8% to 8.7% in GA samples, with no significant difference in these rates between specimens.

### Yield of 2 Versus 1 IS Sample in *Mtb* Culture

Twelve (1.7%) culture-confirmed pulmonary tuberculosis cases were identified among 719 children who had ≥2 IS samples with available culture results. Of the 12 *Mtb* culture-confirmed cases based on IS samples, 2 (16.7%) had positive cultures from both the first and second IS samples, 6 (50.0%) were detected only from the first sample, and 4 (33.3%) only from the second sample. The first IS sample identified 66.7% (95% CI, 34.9%–90.1%) of all culture-confirmed pulmonary tuberculosis cases detected with any IS sample, and the second IS sample identified 50.0% (95% CI, 21.1%–78.9%) (comparison 1 in Figure 1).

### Yield of IS Versus GA Samples in *Mtb* Culture

IS samples identified 8 (42.1%) of the 19 culture-confirmed pulmonary tuberculosis cases identified among patients who had both ≥1 IS and ≥1 GA sample collected (Table 1). In head-to-head comparisons of single IS and GA samples and double IS and GA samples, GA samples alone identified a consistently greater proportion (6 of 11 [54.5%] for one GA sample, and 7 of 12 [58.3%] for two GA samples) of culture-confirmed pulmonary tuberculosis cases than did IS samples alone (3 of 11 [27.3%] for one IS sample, and 2 of 12 [16.7%] for two IS samples) (comparisons 2 and 6 in Figure 1), although these findings were not statistically significant. In the comparison of cases with 2 IS and 2 GA samples, among whom there were 12 with culture-confirmed pulmonary tuberculosis (bolded cells in Table 1), the first IS sample identified 4 cases (33.3%), and the 8 remaining cases (66.7%) were identified through GA sample culture, with or without culture of the second IS sample. The *Mtb* yields were greatest (3.7% and 4.0%, respectively) when 2 GA samples were combined with 1 or 2 IS samples (comparisons 3 and 6 in Figure 1).

### Culture-Confirmed Tuberculosis Identified in Children With No GA Samples Collected

Eight (29.6%) of the 27 culture-confirmed pulmonary tuberculosis cases occurred among children in whom no GA samples were collected, and who were therefore not included in the yield analyses involving GA samples, described above. Five children had diagnoses based on IS samples alone, 2 had positive endotracheal endotracheal tube aspirate samples, and 1 had a positive *Mtb* blood culture (Table 1).

## DISCUSSION

In this study, conducted in a high-burden tuberculosis setting with high HIV prevalence, we demonstrate that a second IS sample was able to identify 50% more pulmonary tuberculosis cases than would have been identified if relying on the submission of a single IS sample (comparison 1 in Figure 1). Among a subset of children who were investigated for pulmonary tuberculosis by ward clinicians through submission of GA samples, we found that GA samples had a higher yield of *Mtb* by culture than IS samples for diagnosing culture-confirmed pulmonary tuberculosis in children <5 years of age hospitalized with WHO severe or very severe pneumonia. This contrasts with the findings of others that IS samples provide a higher yield of *Mtb* culture positivity in children with suspected pulmonary tuberculosis [9]. Furthermore, we showed that a single IS sample underestimates the prevalence of culture-confirmed pulmonary tuberculosis by 67%, compared with the combination of 2 IS and ≥2 GA samples.

The yield of a single IS sample was similar to that of the second IS sample in our study (67% and 50%, respectively; comparison 1 in Figure 1), but both yields were greater than those observed in a large survey of the utility of IS sampling in the diagnosis of pediatric pulmonary tuberculosis in the ambulatory setting; in that study, the yields of the first and second IS samples were 38% and 27%, respectively [30].

Our study demonstrates a 3% prevalence of culture-confirmed *Mtb* in children hospitalized with WHO-defined severe or very severe pneumonia. This is lower than in previous South African studies, in which up to 8% of children hospitalized with acute severe community-acquired pneumonia, with or without hypoxia, had culture-confirmed pulmonary tuberculosis [14, 15]. Significant advancements have been made in child healthcare policy in South Africa since the mid-1990s, which may explain why culture-confirmed *Mtb* is now less prevalent in young children with community-acquired pneumonia in our setting. Advances in prevention of mother-to-child transmission of HIV [33, 34] as well as earlier detection of pediatric HIV infection and scale-up of pediatric antiretroviral therapy [35], have contributed to declines in culture-confirmed *Mtb* in South Africa [21, 36]. Furthermore, there might be a lower force of infection of *Mtb* in South African children, with the ongoing decline in incidence of tuberculosis among adults since 2009 [36].

One or 2 IS specimens and 1–3 GA specimens submitted for *Mtb* culture were positive in 9% and 14% of the children with a clinical or culture-confirmed tuberculosis diagnosis in our study, which is toward the lower estimates of test positivity (7%–43%) in previous reports of children with clinically suspected pulmonary tuberculosis [6, 37]. The extent of lung disease in children with classic pulmonary tuberculosis symptoms may be greater than that occurring in children with primary tuberculosis and concomitant bacterial pneumonia presenting with acute symptoms, making it difficult to draw direct comparisons about sample yields for pulmonary tuberculosis between studies. However, limited ability to establish a culture-confirmed diagnosis of pulmonary tuberculosis in young children reemphasizes the pressing need for more accurate diagnostic assays for childhood tuberculosis, which would also decrease the potential for misdiagnosing the condition and subjecting children to an unnecessary course of antituberculosis treatment.

Limitations of our study include the small number of culture-confirmed pulmonary tuberculosis cases in our cohort, the different manners in which the first and second IS samples were handled in the study, and the selective collection of GA samples. First IS samples were aliquoted for other microbiologic testing in addition to culture for *Mtb*, but the second IS samples were submitted for *Mtb* culture only. This could have reduced the sensitivity of the first IS sample in detecting *Mtb* because of the lower sample volume for *Mtb* culture, although the utility of the second sample in detecting *Mtb* may have been compromised by the increased contamination rates observed in the second IS samples. Children from whom GA samples were collected were significantly more likely to have clinical characteristics associated with tuberculosis disease. If GA samples were routinely collected during the PERCH study, rather than just at the discretion of attending clinicians, the relative contributions of GA and IS samples to culture-confirmed pulmonary tuberculosis diagnosis in the cohort could have been more fully evaluated. Nevertheless, when limiting the comparison of GA and IS samples only to children in whom both specimen types were collected, the yield of *Mtb* culture positivity from GA samples was higher than that from IS samples.

In high-burdened settings of tuberculosis and HIV, children 1–59 months of age presenting with acute community-acquired pneumonia have an appreciable burden of culture-confirmed pulmonary tuberculosis that can be diagnosed through submission of GA and IS samples. Considering that the sensitivity of culture for childhood pulmonary tuberculosis is only 30%–40% across studies [38], it is possible that up to 10% of children hospitalized with acute pneumonia in our setting have underlying pulmonary tuberculosis. The GA and IS sampling techniques complement one another, and a strategy in which 1 or 2 IS and 2 GA samples are submitted on separate days may improve the diagnosis of pulmonary tuberculosis in such patients.

## Supplementary Data

Supplementary materials are available at *Clinical Infectious Diseases* online. Consisting of data provided by the authors to benefit the reader, the posted materials are not copyedited and are the sole responsibility of the authors, so questions or comments should be addressed to the corresponding author.

## Supplementary Material

DAP_16_Submission_to_CID_supplementary_material_24Oct2016

## Notes

***Author contributions.*** D. P. M. performed analyses and drafted manuscripts. M. M. H. assisted with analyses and drafting of the manuscript. L. L. H., M. D .K., D. R. F., D. R. M., K. L. O., and S. A. M. provided guidance on analysis and manuscript. S. A. M. was the principal investigator of the South Africa PERCH study site. P. D. S., V. L. B., and P. V. A. performed laboratory testing. A. M. performed data collection throughout the study. C. P. and A. N. D. assisted with study coordination and manuscript development.

***Acknowledgments.*** We acknowledge the work of the PERCH Study Group and contributors, the PERCH Expert Group, the Pneumonia Methods Working Group, and the PERCH Chest Radiograph Reading Panel. See Supplemental Data for the list of members in each group. We offer sincere thanks to the patients and families who participated in this study. This article is published with the permission of the Director of the Kenya Medical Research Institute.

***Financial support.*** This work is supported by the Bill & Melinda Gates Foundation (grant 48968 to the International Vaccine Access Center, Department of International Health, Johns Hopkins Bloomberg School of Public Health).

***Supplement sponsorship.*** This article appears as part of the supplement “Pneumonia Etiology Research for Child Health (PERCH): Foundational Basis for the Primary Etiology Results,” sponsored by a grant from the Bill & Melinda Gates Foundation to the PERCH study of Johns Hopkins Bloomberg School of Public Health, Baltimore, Maryland.

***Potential conflict of interest.*** M. D. K. has received funding for consultancies from Merck, Pfizer, and Novartis, and grant funding from Merck. L. L. H. has received grant funding from Pfizer and GlaxoSmithKline (GSK). S. A. M. has received honoraria for advisory board participation from Bill & Melinda Gates Foundation (BMGF), Pfizer, Medimmune, and Novartis and institutional grants from GSK, Novartis, Pfizer, Minervax, and BMGF and has served on the speakers bureau for Sanofi Pasteur and GSK. K. L. O. has received grant funding from GSK and Pfizer and participates on technical advisory boards for Merck, Sanofi Pasteur, PATH, Affinivax, and ClearPath. All other authors: No reported conflicts. All authors have submitted the ICMJE Form for Disclosure of Potential Conflicts of Interest. Conflicts that the editors consider relevant to the content of the manuscript have been disclosed.
