# Supplementary material for: The Incremental Value of Repeated Induced Sputum and Gastric Aspirate Samples for the Diagnosis of Pulmonary Tuberculosis in Young Children With Acute Community-Acquired Pneumonia
Source: Clin Infect Dis. 2017 May 27;64(Suppl 3):S309–16. doi: 10.1093/cid/cix099 (PMC5447846; doi:10.1093/cid/cix099)
Supplement: DAP_16_Submission_to_CID_supplementary_material_24Oct2016 [file cix099_suppl_DAP_16_Submission_to_CID_supplementary_material_24Oct2016.pdf]

Supplementary Figure 1. Number of Induced Sputum (IS) and Gastric Aspirate (GA) Samples Available for Mycobacterial Culture Yield Analyses

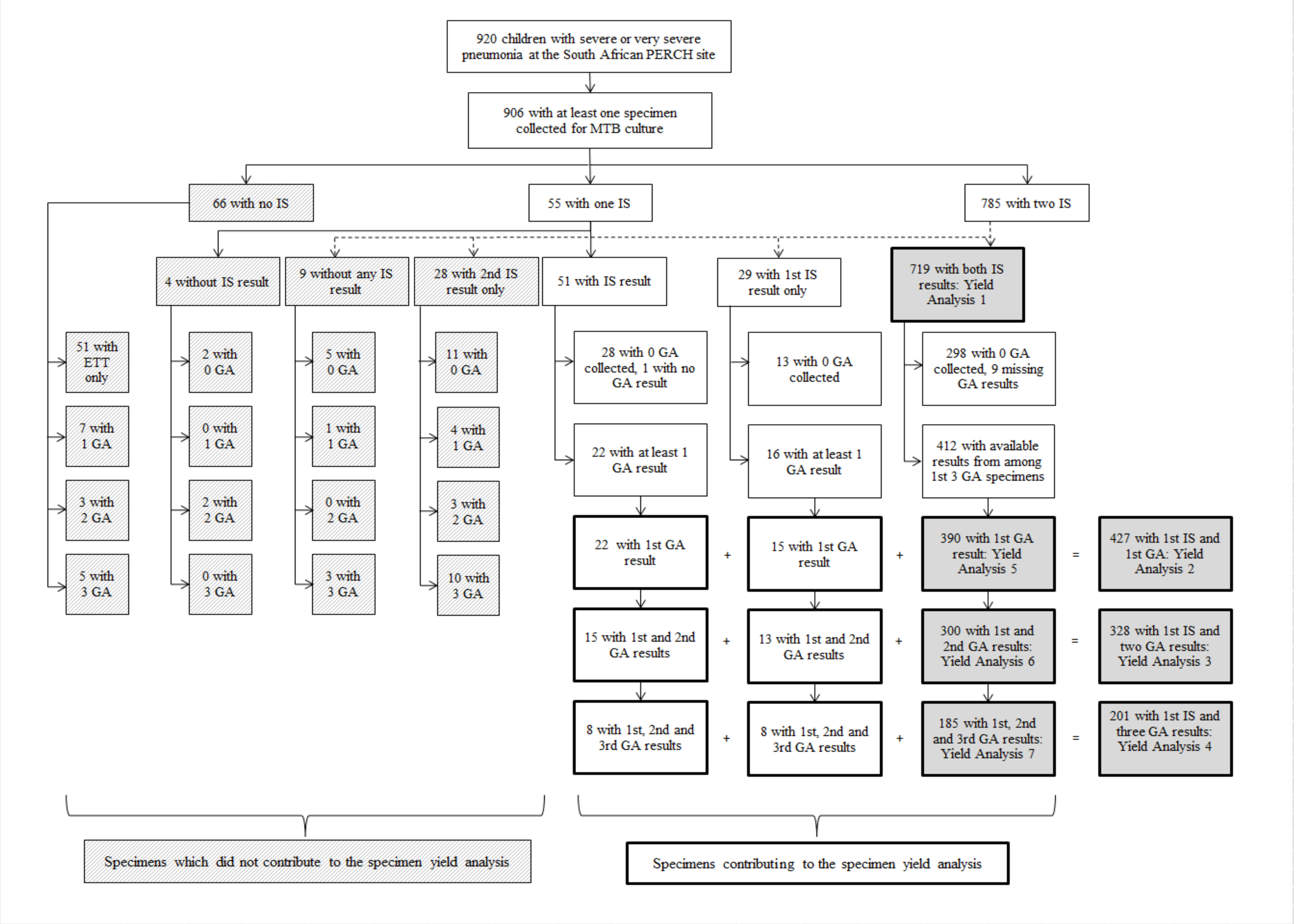

Supplementary Table 1. Characteristics of Cases with and without Gastric Aspirates (GA) Collected

	**GA Collected (N=498)**		**No GA collected (N=422)**		**P-value[a]**	**Odds Ratio (95%CI)[b]**	**Adjusted Odds Ratio (95%CI)[c]**
	**n**	**%**	**n**	**%**			
Age Group							
1-5 months	223	44.8	235	55.8	.003	Ref	Ref
6-11 months	140	28.1	85	20.2		1.74 (1.25 to 2.40)	3.36 (1.45 to 7.75)
12-23 months	94	18.9	63	15.0		1.57 (1.09 to 2.27)	3.05 (1.14 to 8.15)
24-59 months	41	8.2	38	9.0		1.14 (0.71 to 1.83)	20.76 (1.98 to 217.20)
Sex							
Female	230	46.2	204	48.5	.49	0.91 (0.70 to 1.19)	
Male	268	53.8	217	51.5		Ref	
Severity							
Severe pneumonia	361	72.5	260	61.8	.001	Ref	
Very severe pneumonia	137	27.5	161	38.2		0.61 (0.46 to 0.82)	
Died							
Died in hospital	12	2.4	26	6.2	.004	0.38 (0.17 to 0.78)	
Died within 30 days[d]	16	3.7	33	8.9	.003	0.41 (0.21 to 0.79)	
HIV infection	80	16.1	35	8.3	<.001	2.11 (1.36 to 3.31)	
Pneumonia severity signs							
Lower chest wall indrawing	482	96.8	399	94.8	.12	1.66 (0.82 to 3.43)	
Head nodding	118	23.7	128	30.4	.02	0.71 (0.52 to 0.96)	
Unable to feed/vomiting everything	12	2.4	32	7.6	<.001	0.30 (0.14 to 0.61)	
Central cyanosis	7	1.4	11	2.6	.19	0.53 (0.17 to 1.52)	
Lethargy	17	3.4	35	8.3	.001	0.39 (0.20 to 0.73)	
Multiple or prolonged convulsions	3	0.6	9	2.1	.08	0.28 (0.05 to 1.12)	
Hypoxemia[e]	367	73.8	323	77.3	.23	0.83 (0.61 to 1.13)	
Tachypnea[f]	396	80.5	308	75.3	.06	1.35 (0.99 to 1.86)	

Supplementary Table 1. Characteristics of Cases with and without Gastric Aspirates (GA) Collected (*continued*)

	**GA Collected (N=498)**		**No GA collected (N=422)**		**P-value[a]**	**Odds Ratio (95%CI)[b]**	**Adjusted Odds Ratio (95%CI)[c]**
	**n**	**%**	**n**	**%**			
Chest radiograph							
Normal	125	29.2	133	37.7	.01	Ref	
Abnormal[g]	303	70.8	220	62.3		1.47 (1.07 to 2.00)	3.36 (1.45 to 7.75)
Malnutrition (weight for age z-score)							
Normal	347	69.8	294	70.7		Ref	
Moderate	75	15.1	51	12.3	.39	1.25 (0.85 to 1.84)	
Severe	75	15.1	71	17.1		0.89 (0.62 to 1.28)	
History of symptoms							
Fever	344	69.1	243	57.7	<.001	1.64 (1.24 to 2.17)	
Cough	485	97.4	403	96	.22	1.57 (0.71 to 3.57)	
Difficulty breathing	458	92.2	403	95.7	.03	0.52 (0.28 to 0.96)	
Wheeze	147	29.6	115	27.4	.45	1.12 (0.83 to 1.51)	
Weight loss/Inability to gain weight	96	19.4	56	13.4	.02	1.55 (1.07 to 2.26)	
Chronic symptoms (>=14 days)							
Fever	3	0.9	2	0.9	1	1.06 (0.12 to 12.78)	
Cough	28	5.9	10	2.5	.02	2.42 (1.12 to 5.66)	
Difficulty breathing	8	1.8	3	0.8	.17	2.43 (0.58 to 14.30)	
Wheeze	4	3.1	1	0.9	.38	3.31 (0.32 to 164.36)	
Any of above	32	6.6	12	2.9	.01	2.35 (1.16 to 5.07)	
Clinical signs on admission							
Stridor	15	3.0	33	7.9	.001	0.37 (0.18 to 0.71)	
Grunting	32	6.5	28	6.7	.89	0.96 (0.55 to 1.70)	
Nasal flaring	409	82.1	359	85.9	.12	0.76 (0.52 to 1.10)	0.15 (0.04 to 0.50)
Deep breathing	63	12.7	73	17.4	.046	0.69 (0.47 to 1.01)	

Supplementary Table 1. Characteristics of Cases with and without Gastric Aspirates (GA) Collected (*continued*)

	**GA Collected (N=498)**		**No GA collected (N=422)**		**P-value[a]**	**Odds Ratio (95%CI)[b]**	**Adjusted Odds Ratio (95%CI)[c]**
	**n**	**%**	**n**	**%**			
Tuberculosis (TB) Medication							
TB Meds started on day of admission	7	1.4	16	3.8	.02	0.36 (0.13 to 0.95)	0.01 (0.00 to 0.34)
On TB Meds at discharge	91	22.3	20	5.6	<.001	4.85 (2.88 to 8.50)	
Positive tuberculin skin test[h]	49	14.9	9	6	.01	2.74 (1.28 to 6.52)	15.45 (2.86 to 83.39)
Duration of Symptoms (days)	**median**	**IQR**	**median**	**IQR**			
Fever	2	1 to 3	2	1 to 3	.11	1.08 (0.98 to 1.19)	
Cough	3	2 to 4	3	2 to 5	.002	1.04 (1.01 to 1.08)	
Difficulty breathing	2	1 to 3	2	1 to 3	.001	1.08 (1.01 to 1.15)	1.78 (1.24 to 2.56)
Wheeze	2	1 to 3	2	1 to 3	.28	1.06 (0.96 to 1.17)	
Length of hospitalization (days)	7	4 to 10	6	4 to 10	.18	0.99 (0.98 to 1.00)	

[a] P-values were obtained using Wilcoxon Rank Sum for continuous variables with skewed distributions, Chi-square for categorical variables and Fishers exact test for categorical variables with fewer than five expected observations in two-by-two tables.
[b] Odds ratios and adjusted odds ratios reflect the odds of gastric aspirate being collected compared to not being collected.
[c] aOR presented for variables included in the final model obtained by performing forward stepwise logistic regression which took into consideration the effects of age at enrolment, clinical history (including failure to gain weight, duration of cough, fever or difficulty breathing and chronic symptomatology), HIV status, pneumonia severity signs on admission (lower chest wall indrawing, head nodding, inability to feed, central cyanosis and lethargy), pneumonia severity categorization, hypoxia on admission, clinical signs on admission (stridor, nasal flaring, deep breathing), timing of initiation of anti-tuberculosis medications, chest X-ray abnormalities, Mantoux response, duration of hospitalization and in-hospital death.
[d] Restricted to 715 children with known vital status at follow-up visit.
[e] Hypoxemia defined as oxygen saturation < 90% on room air, or on supplemental oxygen if no room air reading available.
[f] Tachypnea defined as ≥60 breaths per minute (bpm) for children <2 months of age, ≥50 bpm for children 2-11 months, and ≥40 bpm for children 12-59 month.
[g] Chest radiographs were deemed to be abnormal if alveolar consolidation, other infiltrate, or both were observed on films obtained within 72 hours of hospitalization.
[h] Restricted to 479 children with available Mantoux results.

Acknowledgements

PERCH Study Group. **Johns Hopkins Bloomberg School of Public Health, Baltimore, Maryland**: Katherine L. O'Brien (PI), Orin S. Levine (Former PI, current affiliation Bill & Melinda Gates Foundation, Seattle, Washington**),** Maria Deloria Knoll (co-PI), Daniel R. Feikin (joint affiliation with Centers for Disease Control and Prevention, Atlanta, Georgia), Andrea N. DeLuca, Amanda J. Driscoll, Nicholas Fancourt, Wei Fu, Laura L. Hammitt, Melissa M. Higdon, E. Wangeci Kagucia, Ruth A. Karron, Mengying Li, Daniel E. Park, Christine Prosperi, Zhenke Wu, Scott L. Zeger; **The Emmes Corporation, Rockville, Maryland:** Nora L. Watson, **Nuffield Department of Clinical Medicine, University of Oxford, United Kingdom**: Jane Crawley; **University of Otago, Christchurch, New Zealand**: David R. Murdoch; **Respiratory and Meningeal Pathogens Research Unit, University of the Witwatersrand , Johannesburg, South Africa**: Shabir A. Madhi (site PI), David P. Moore, Peter V. Adrian, Vicky L. Baillie, Locadiah Kuwanda, Azwifarwi Mudau, Michelle J. Groome, Nasreen Mahomed.

PERCH Expert Group. William C. Blackwelder, Harry Campbell, John A. Crump, Adegoke Falade, Menno D de Jong, Claudio Lanata, Kim Mulholland, Shamim Qazi, Cynthia G. Whitney.

Pneumonia Methods Working Group. Robert E Black, Zulfiqar A Bhutta, Harry Campbell, Thomas Cherian, Derrick W Crook, Menno D de Jong, Scott F Dowell, Stephen M Graham, Keith P Klugman, Claudio F Lanata, Shabir A Madhi, Paul Martin, James P Nataro, Franco M Piazza, Shamim A Qazi, and Heather J Zar.

PERCH Chest Radiograph Reading Panel

Readers: Dr. Kamrun Nahar, Dr. Fariha Bushra Matin, Dr. Claire Oluwalana, Dr. Bernard Ebruke, Dr. Joyce Sande, Dr. Micah Silaba Ominde, Dr. Mahamadou Diallo, Dr. Breanna Barger-Kamate, Dr. Nasreen Mahomed, Dr. David P. Moore, Dr. Anchalee Kruatrachue, Dr. Piyarat Suntarattiwong, Dr. Musaku Mwenechanya, Dr. Rasa Izadnegahdar, **Arbitrators:** Dr. Vera Manduku, Dr. John DeCampo, Dr. Marg DeCampo, Dr. Fergus Gleeson.

PERCH Contributors:

South Africa: Azwidihwi Takalani, Andrea Hugo, Susan Nzenze, Ndulela Titi, Mmabatho Selela, Malebo Motiane, Minah Nkuna, Nonhlanhla Tsholetsane, Sibonsile Moya, Debra Katisi, Tondani Netshishivhe, Lerato Mapetla, Gudani Singo, Simphiwe Gasa, Cece Mgenge, Nozipho Mthunzi, Nombulelo Monedi, Tanja Adams, Shafeeka Mangera, Jeannette Wadula, Peter Tsaagane, Jenifer L. Vaughan, Sakina Loonat, Martin Hale, Sugeshnee Pather, Mariëtte Middel, Siobhan Trenor, Palesa Morailane, Ntombi Maya, Rene Sterley, Charné Combrinck, Given Malete, Lerato Qoza, Grizelda Liebenberg, Hendrik van Jaarsveld,

Zunaid Kraft, Lisa-Marie Mollentze, Lourens Combrinck, Tsholofelo Mosome; **Canterbury Health Laboratory, Christchurch, New Zealand:** Trevor P. Anderson, Joanne Mitchell, Rose Watt, Shalika Jayawardena; **The Emmes Corporation, Rockville, Maryland:** Mark Wolff, Megan Sanza, Omid Neyzari.
